# On the impovesrishment of scientific education

**DOI:** 10.1186/1687-4153-2013-15

**Published:** 2013-11-11

**Authors:** Edward R Dougherty

**Affiliations:** 1 Center for Bioinformatics and Genomic Systems Engineering, Department of Electrical and Computer Engineering, Texas A&M University, 3128 TAMU, College Station, TX 77843-3128, USA

## Abstract

Hannah Arendt, one of the foremost political philosophers of the twentieth century, has argued that it is the responsibility of educators not to leave children in their own world but instead to bring them into the adult world so that, as adults, they can carry civilization forward to whatever challenges it will face by bringing to bear the learning of the past. In the same collection of essays, she discusses the recognition by modern science that Nature is inconceivable in terms of ordinary human conceptual categories - as she writes, ‘unthinkable in terms of pure reason’. Together, these views on scientific education lead to an educational process that transforms children into adults, with a scientific adult being one who has the ability to conceptualize scientific systems independent of ordinary physical intuition. This article begins with Arendt’s basic educational and scientific points and develops from them a critique of current scientific education in conjunction with an appeal to educate young scientists in a manner that allows them to fulfill their potential ‘on the shoulders of giants’. While the article takes a general philosophical perspective, its specifics tend to be directed at biomedical education, in particular, how such education pertains to translational science.

## Review

### Introduction

*Between Past and Future* is a collection of essays written by Hannah Arendt between 1954 and 1968 in which, among many other issues, she makes basic points regarding education and science that when taken together entail a certain kind of scientific education [1]. From a general perspective, education should provide students with the knowledge to renew the world, that is, to refresh and keep vibrant a civilization that, except for the ability to take on new and unforeseen crises, would succumb to the vicissitudes of Nature and the human condition.

In *The Crisis in Education*, Arendt writes,

Education is the point at which we decide whether we love the world enough to assume responsibility for it and by the same token save it from that ruin which, except for renewal, except for the coming of the new and young, would be inevitable. And education, too, is where we decide whether we love our children enough not to expel them from our world and leave them to their own devices, nor to strike from their hands their chance of undertaking something new, something unforeseen by us, but to prepare them in advance for the task of renewing a common world [2].

Sound education is not an option; society depends upon it, for otherwise humans would lack the capacity to renew their world since, in an endless cycle, the old must pass on and leave it to the young to carry on. This places a heavy responsibility upon educators. They must not leave the young to fend for themselves; rather, they must provide them with the fundamentals required to maintain and extend human knowledge. These fundamentals apply not only to a specialized field of research; they encompass a wide range of learning across many fields, including science, mathematics, philosophy, and history. Significant scientific knowledge does not rest on particular technical relationships alone but rather on the integration of myriad philosophical-scientific sources that facilitate deep conceptualizations. Absent the ability to conceptualize, one cannot engage in the creative thought needed to discover new knowledge and address the crises lurking in the future. To provide the young with the fundamentals necessary for renewal, the educator must transform children into adults.

### Nature is unthinkable

For aspiring young scientists, this transformation is governed by the nature of scientific knowledge. This leads us to Arendt’s basic point regarding scientific knowledge. In *The Conquest of Space and the Stature of Man*, she writes,

To understand physical reality seems to demand not only the renunciation of an anthropocentric or geocentric world view, but also a radical elimination of all anthropomorphic elements and principles, as they arise either from the world given to the five senses or from the categories inherent in the human mind [3].

In a similar vein, in *The Concept of History: Ancient and Modern*, she writes,

The trouble, in other words, is not that the modern physical universe cannot be visualized, for this is a matter of course under the assumption that Nature does not reveal itself to the human senses; the uneasiness begins when Nature turns out to be inconceivable, that is, unthinkable in terms of pure reasoning as well [4].

Not only need we reject an anthropocentric world view, as one might do when accepting the Copernican hypothesis, and not only need we accept the inability of our senses to reveal Nature in her true form, but much more than either of these limitations, we must accept that Nature is so strange to us that it is not even thinkable in terms of the human categories of understanding.

It became quite clear in the first half of the twentieth century, with the advent of the quantum theory and general relativity, that ideas such as particle, wave, and force, whose origins lay in pre-scientific perceptual experience, and frames of reference, such as Euclidean three-dimensional space and linear time, and underlying hypotheses concerning regularity, such as causality and continuity, were inadequate, or even detrimental, to scientific conceptualization. Erwin Schrodinger puts the matter this way:

As our mental eye penetrates into smaller and smaller distances and shorter and shorter times, we find nature behaving so entirely differently from what we observe in visible and palpable bodies of our surrounding that no model shaped after our large-scale experiences can ever be ‘true’. A completely satisfactory model of this type is not only practically inaccessible, but not even thinkable. Or, to be precise, we can, of course, think it, but however we think it, it is wrong; not perhaps quite as meaningless as a ‘triangular circle’, but much more so than a ‘winged lion’ [5].

Because science concerns relations between measurable variables and it is these relations that constitute the subject matter of science, scientific knowledge *ipso facto* is mathematically constituted. Nonetheless, scientists had historically attached physical descriptions in the sense of our ordinary categories of understanding to their mathematical systems; however, once it is recognized that the behavior of the phenomena is unthinkable in terms of our ordinary pre-scientific categories, such descriptions are no longer satisfactory. While they might be useful in organizing one’s thinking, they are superfluous; indeed, they can be misleading. In the words of James Jeans, ‘The final truth about phenomena resides in the mathematical description of it; so long as there is no imperfection in this, our knowledge is complete. We go beyond the mathematical formula at our own risk’ [6]. Scientific knowledge is not constrained by the limitations of human physical understanding developed in our everyday world of experience; indeed, everyday physical thinking can be an impediment to scientific knowledge. Lack of sensible experience applies not only to the quantum world; it also applies to complexity. Humans have no perceptual experience with systems, such as cells, involving hundreds of thousands of interacting components. The mind boggles and intuition crashes when confronted with such immense complexity.

Whatever the reason might be for our inability to think about Nature *qua* Nature, human knowledge of Nature is not limited by our understanding of Nature. Arendt writes, ‘What defies description in terms of the “prejudices” of the human mind defies description in every conceivable way of human language; it can no longer be described at all, and it is being expressed, but not described, in mathematical processes’ [3]. The prejudices to which she refers are, in the words of Niels Bohr, the categories of ‘our necessarily prejudiced conceptual frame’, [7] and include categories such as causality and determinism. In sum, our scientific knowledge of Nature is not given by description of the phenomena; rather, it is constituted by mathematical processes.

As for the validity (or ‘truth’) of such processes, the sole criterion is their functionality as predictors of future behavior. This means that a scientific theory consists of two parts: (1) the mathematical theory itself and (2) a set of relations, called *operational definitions*, that connects the theory to the phenomena so that the predictive capacity of the theory can be tested. The operational definitions themselves are mathematical in form since the accuracy of prediction must be understood within the framework of statistics. Without going into detail on the statistical issues involved, the key point in the present exposition is that the mathematical theory must be formally connected to future observations and the firmness of this connection determines the truth of the theory. One’s personal predilections, such as a metaphysical belief in causality, play no role in the acceptance or rejection of a theory. Richard Feynman writes, ‘It is whether or not the theory gives predictions that agree with experiment. It is not a question of whether a theory is philosophically delightful, or easy to understand, or perfectly reasonable from the point of view of common sense’ [8]. The latter point is crucial. As stated by Arendt, Nature is ‘unthinkable in terms of pure reasoning’, so reasoned arguments can play no role in validating a scientific theory.

The most remarkable aspect of modern science is that when confronted with the inability to understand Nature, human beings do not stand helpless before Nature. Modern science has turned away from the ancient and medieval attempts to describe Nature to building mathematical systems that can predict phenomena. Arendt writes, ‘Man can *do*, and successfully do, what he cannot comprehend and cannot express in everyday human language’ [3]. That is, although the theory cannot be put into words, given the operational definitions, it can be tested. Mathematical reasoning allows us to go beyond our physical reasoning in characterizing phenomenal relations. Historian Morris Kline writes, ‘What science has done, then, is to sacrifice physical intelligibility for the sake of mathematical description and mathematical prediction’ [9]. Notice that Kline refers to ‘mathematical description’, not description in terms of the ‘prejudices’ of the human mind. Intelligibility resides in these ‘prejudices’, and therefore, we should not expect scientific theories to be intelligible. This is why Feynman says, ‘I hope you can accept Nature as she is — absurd’ [8]. Indeed, Nature is *ipso facto* absurd because it is unintelligible.

While it may be true that our inability to think about Nature in terms of our ordinary categories of physical understanding has been brought into clear focus on account of general relativity and the quantum theory, as Arendt points out, the issue has been with us since the dawn of modern science in the seventeenth century. In *Dialogues Concerning Two New Sciences*, Galileo puts these words into the mouth of Salviati:

The present does not seem to me to be an opportune time to enter into the investigation of the cause of the acceleration of natural motion… For the present, it suffices our Author that we understand him to want us to investigate and demonstrate some attributes of a motion so accelerated (whatever be the cause of its acceleration) that the momenta of its speed go increasing, after its departure from rest, in that simple ratio with which the continuation of time increases, which is the same as to say that in equal times, equal additions of speed are made [10].

Galileo does not deny causality; he simply *brackets* it (puts it aside), ignores it, and gets on with the business of obtaining mathematical relations between phenomena. He writes that there would be ‘little gain’ in examining the kind of ‘fantasies’ put forth by philosophers to explain acceleration in terms of causality. It is more beneficial to ‘investigate and demonstrate some attributes of motion’. Although Galileo does not deny causality, as opposed to Aristotle, he rejects it as a requirement for knowledge.

Like Galileo, Newton believes in causality but brackets it outside of science. Near the beginning of *The Principia: Mathematical Principles of Natural Philosophy*, he writes, ‘For I here design only to give a mathematical notion of these forces, without considering their physical causes and seats’ [11]. Near the end of *The Principia*, he states, ‘Hitherto I have not been able to discover the cause of those properties of gravity from the phenomena, and I frame no hypothesis; for whatever is not deduced from the phenomena is to be called an hypothesis; and hypotheses, whether metaphysical or physical, whether of occult qualities or mechanical, have no place in experimental philosophy’ [11].

Ancient and medieval science comes to an end with Galileo and Newton, who are thoroughly modern in recognizing that human language and the concepts constructed within that language are not sufficient for science. Kline writes, ‘The insurgent seventeenth century found a qualitative world whose study was aided by mathematical abstractions. It bequeathed a mathematical, quantitative world that subsumed under its mathematical laws the concreteness of the physical world’ [9].

It would be David Hume who would fully comprehend that causality cannot be logically or empirically deduced from natural phenomena and therefore is not a scientific category. He notes that a cause and its effect are contiguous and related via temporal priority, with the cause prior to the effect. But causality corresponds to more than contiguity and temporal priority; it relates to a ‘necessary connection’ between the cause and the effect. However, the principle of causality is neither intuitively certain nor provable by logical means, and according to Hume, our belief in the principle rests not on reason, but on habit and custom.

Immanuel Kant agrees with Hume that the principle of causality is not a scientific principle; however, whereas for Hume, habit underlies belief in causality, for Kant, causality is a category of understanding that imposes forms on the data of sensation, and scientific knowledge is limited by these forms. The way things appear, such as being spatially coordinated and connected by causality, is due to subjective *a priori* conditions for human knowledge. One cannot know things apart from the manner in which they conform to these *a priori* mental forms. While Kant differs from Hume on the ground of causality, regarding Nature, the basic point remains. Kant writes, ‘[Hume] justly maintains that we cannot comprehend by reason the possibility of causality, that is, of the reference of the existence of one thing to the existence of another, which is necessitated by the former’ [12]. Rather, causality is automatically imposed upon the phenomena to make them thinkable.

Even if this is so, why should they be thinkable? Jeans states the matter concisely: ‘We need no longer discuss whether light consists of particles or waves; we know all there is to be known about it if we have found a mathematical formula which accurately describes its behavior, and we can think of it as either particles or waves according to our mood and the convenience of the moment’ [6]. One can think about the phenomena in terms of ordinary physical categories of understanding, but the choice of how one chooses to think about them depends on one’s predilections of the moment. The danger is that the intuitions associated with the phenomena might have nothing to do with them - or worse, be completely misleading.

### Educational implications

Two major educational implications arise from the inconceivability of Nature. One is technical and relates to the ability to conceptualize and hence form scientific theories. The other is more general and has to do with appreciating and working within an epistemology that presupposes conceptualizations outside those of the ordinary understanding and outside of ordinary language.

#### Technical implications

Broad mathematical knowledge gives a scientist greater capability for conceptualization. Thus, it is obvious that budding scientists should be armed with a large mathematical tool box rich in the mathematics appropriate to one’s field - the deeper the mathematical knowledge, the more suitable for framing fundamental scientific knowledge. For instance, since the characterization of cellular behavior involves massive stochastic systems of interacting genes and proteins, to constitute biological knowledge at more than a superficial level, a biologist must be armed with a working knowledge of stochastic processes. How else would it be possible to conceptualize the processes that will inevitably constitute the theory characterizing signaling pathways within the cell? This does not simply mean that a biologist needs to possess a mathematical understanding of existing stochastic models for cellular behavior; more to the point, being a biologist means to formulate scientific theories, not simply to read about the theories of others. The biologist must also formulate experiments that elicit relevant behavior of cellular pathways and use the resulting observations to formulate network models. The need for a rich tool box is not an esoteric requirement for a small group of theoretical academicians; it applies directly to those studying regulatory diseases such as cancer.

None of this is new. In 1948, Norbert Wiener wrote, ‘The group of scientists about Dr. Rosenblueth and myself had already become aware of the essential unity of the set of problems centering about communication, control, and statistical mechanics, whether in the machine or in living tissue’ [13]. By 1948, Wiener had recognized the epistemological unity of systems-based sciences, be they electrical, economic, or biological systems. The fact that biology concerns systems was noted in 1935 by Conrad Waddington, who wrote, ‘To say that an animal is an organism means in fact two things: firstly, that it is a system made up of separate parts, and secondly, that in order to describe fully how any one part works one has to refer either to the whole system or to the other parts’ [14].

Today, given the vast body of relevant knowledge accumulated since the 1930s, would it not behoove our educational system to educate biologists so that they possess a working knowledge of stochastic systems? What could possibly be the point of not educating biologists so that they have the knowledge to address biological problems at a deep level? Why send out young researchers to try to engineer solutions to cancer without first educating them in the well-established theory of stochastic control? As M. L. Bittner and I have written elsewhere, ‘Isaac Newton published his *Principia* in 1646; Pierre-Simon Laplace published the first volume of his *Celestial Mechanics* 150 years later in 1796. Laplace's system depends on the calculus of Newton and its subsequent developments over a century and a half. Laplace did not ignore the well-developed mathematics of his day and try to develop his mechanics without it; rather, he used the relevant available tools’ [15]. Is there any rationale for sending young scientists out into the research world with the vain hope that elementary mathematics will suffice for the investigation of complex regulatory networks? We return to Arendt’s first point: Do ‘we love our children enough not to…leave them to their own devices?’

This is not to argue that biologists or physicists need to be mathematicians. Albert Einstein was not a mathematician and had the assistance of a number of outstanding and great mathematicians, including David Hilbert. What Einstein had, however, was sufficient mathematical knowledge to give him the power of conceptualization. He writes, ‘Experience, of course, remains the sole criterion for the serviceability of mathematical constructions for physics, but the truly creative principle resides in mathematics’ [16]. The creative principle must lie in mathematics because scientific theory is conceived in mathematics. While a scientist need not be a mathematician, there is a threshold that must be crossed. Wiener clarifies the issue very well: ‘The mathematician need not have the skill to conduct a physiological experiment, but he must have the skill to understand one, to criticize one, and to suggest one. The physiologist need not be able to prove a certain mathematical theorem, but he must be able to grasp its physiological significance and tell the mathematician for what he should look for’ [13].

Referring to my 2012 book with M. L. Bittner, *Epistemology of the Cell*[17], Terrence McGarty writes, ‘The authors place a stake in the ground to say what would be expected for those to work in the field, that the books by Loeve and Cramer be used as standard bearers!…Thus they set a high hurdle, but a necessary one for those to work in the field’ [18]. Two points can be made regarding McGarty’s comment. First, Michel Loeve published his *Probability Theory* in 1955 and Harald Cramer published his *Mathematical Methods in Statistics* in 1946. Surely more than half a century later, statisticians involved in scientific research should at least be at the level of these seminal books; however, perusal of the bioinformatics literature provides convincing evidence that a large number of recent Ph.D.s lack proficiency in the basics of their subject. The second point regarding McGarty’s comment is that these books set a ‘necessary’ standard for medical research based on cell dynamics. Given that this standard is not being met, can we expect any more from the billions of dollars poured into biomedical research than the meager, and often meaningless or even erroneous, results now being published [19-24]?

The situation is far worse than statisticians not knowing fundamental theory. There is growing evidence that statisticians in major research groups apparently cannot even properly utilize rudimentary statistics. John Ioannidis writes, ‘There is increasing concern that in modern research, false findings may be the majority or even the vast majority of published research claims…. Simulations show that for most study designs and settings, it is more likely for a research claim to be false than true’ [19]. Mehta et al*.* write, ‘Many papers aimed at the high dimensional biology community describe the development or application of statistical techniques. The validity of many of these is questionable, and a shared understanding about the epistemological foundations of the statistical methods themselves seems to be lacking’ [22]. Alain Dupuy and Richard Simon, Chief of the Biometric Research Branch, Division of Cancer Treatment and Diagnosis, of the National Cancer Institute, state, ‘Both the validity and the reproducibility of microarray-based clinical research have been challenged’ [23]. Based on a detailed analysis of 42 studies published in 2004, Dupuy and Simon report that 21 (50%) of them contain at least one of three basic flaws. The situation is actually much worse because, as will shortly be discussed, many use error estimation methods, that while properly computed, are not applicable under the experimental conditions in which they are being employed (Dupuy and Simon only consider an error estimate to be flawed if it is calculated incorrectly). Needless to say, the vast majority of erroneous research findings are favorable to the authors’ claims. This phenomenon has been politely termed ‘over-optimism’ [24]. Anne-Laure Boulesteix writes, ‘The difficulty to publish negative results obviously encourages authors to find something positive in their study by performing numerous analyses until one of them yields positive results by chance, i.e. to fish for significance’ [24].

Lest one think that Ioannidis and Boulesteix, and others, are being overly pessimistic, according to a recent report regarding comments by Janet Woodcock, Director of the Center for Drug Evaluation and Research at the FDA, she has estimated that as much as 75% of published biomarker associations are not replicable. She states, ‘This poses a huge challenge for industry in biomarker identification and diagnostics development’ [25]. Much of the blame for these non-reproducible findings rests with a cavalier attitude towards the application of statistical methods [26].

There are various ways to fish for significance, but the sport often revolves around bogus error estimation: use an error estimation procedure with large variance so that when the analysis is repeated with different data analysis methods, it is highly probable that a good-looking (but phony) result will occur and then report that result. When trying to find sets of genes to classify a disease, two popular fishing methods are to try a number of different data sets [27] or try numerous methods to design the classifier [28]. In the first case, an error estimate is computed for each data set, and in the second, an error estimate is computed for each attempted classifier. Such fishing can be hard to detect unless the authors' reveal how many data sets and classification schemes they have tried.

One should not jump to the conclusion that fishing represents a deliberate attempt to publish fraudulent research; rather, the widespread use of error estimation techniques such as cross-validation makes it much more likely that it is simply a matter of inadequate education. Given that these estimates can be used by a sixth grader and appear in text books absent any proof that they should provide accurate results, and given ample evidence going back to 1978 showing that they should not be expected to produce accurate results when samples are small [29-34] as is very often the case in real-world situations, one can reasonably conclude that society is reaping the rewards of educational impoverishment. One can hardly imagine a statistician brought up on Cramer being so cavalier with statistical methods.

#### Epistemological implications

Science has a rich and varied history. Its epistemological ground has shifted from its totally empirical Egyptian-Mesopotamian beginnings through its integration with metaphysics with Aristotle to the beginnings of the experimental-mathematical duality with Francis Bacon and Galileo and onto complete freedom from conception within the ordinary categories of understanding in the twentieth century. A deep appreciation of this history allows a scientist to see his work in the stream of civilization and to avoid falling into the myriad of fruitless paths that have beguiled our predecessors.

For those who truly wish to be scientists, Einstein’s following words penned in a letter should be taken to heart:

I fully agree with you about the significance and educational value of methodology as well as history and philosophy of science. So many people today – and even professional scientists – seem to me like somebody who has seen thousands of trees but has never seen a forest. A knowledge of the historic and philosophical background gives that kind of independence from prejudices of his generation from which most scientists are suffering. This independence created by philosophical insight is – in my opinion – the mark of distinction between a mere artisan or specialist and a real seeker after truth [35].

It is natural for Einstein to refer to the ‘prejudices of his generation’. As a young man he had broken free from the Newtonian world that had been regnant since Newton’s *Principia*.

Three centuries before Einstein, Francis Bacon urged mankind to break free from prejudices of two millennia when in his *Novum Organum*, he called upon natural philosophers to go beyond haphazard observation of Nature to directed and purposeful observation:

There remains simple experience which, if taken as it comes, is called accident; if sought for, experiment. But this kind of experience is no better than a broom without its band, as the saying is — a mere groping, as of men in the dark, that feel all round them for the chance of finding their way, when they had much better wait for daylight, or light a candle, and then go. But the true method of experience, on the contrary, first lights the candle, and then by means of the candle shows the way; commencing as it does with experience duly ordered and digested, not bungling or erratic, and from it educing axioms, and from established axioms again new experiments [36].

Reflecting on the rapid advance of post-Galilean science in comparison to the scanty achievements of the preceding 2,000 years, in the preface of the second edition of the *Critique of Pure Reason*, Kant writes,

Reason must approach nature with the view, indeed, of receiving information from it, not, however, in the character of a pupil, who listens to all that his master chooses to tell him, but in that of a judge, who compels the witnesses to reply to those questions which he himself thinks fit to propose. To this single idea must the revolution be ascribed, by which, after groping in the dark for so many centuries, natural science was at length conducted into the path of certain progress [37].

As opposed to groping about amid unstructured observations, in accordance with his own mental constructs, the scientist imposes himself upon Nature by setting up conditions that constrain Nature to behave in ways that provide answers to targeted questions.

We hear the reverberation of Bacon and Kant in the words of statistician Douglas Montgomery: ‘By the statistical design of experiments we refer to the process of planning the experiment so that appropriate data will be collected, which may be analyzed by statistical methods resulting in valid and objective conclusions. The statistical approach to experimental design is necessary if we wish to draw meaningful conclusions from the data’ [38]. Statistical experimental design is a key part of the evolution of scientific thinking over the last four centuries. Moreover, the validity of scientific knowledge is characterized by the predictive capacity of a theory, and the predictive capacity must be evaluated using proper statistical theory. Groping and fishing are out of place here.

Contemporary efforts at groping and fishing go under the name of ‘data mining’. Massive amounts of unstructured data are being collected via all sorts of expensive technology without any experimental design. The data set is said to be ‘big’ when it contains an enormous number of measurements. A so-called big data set often arises from measuring tens of thousands of variables with only a small number of replicates. Hence, from a statistical perspective, the data set is extremely small, because the number of replicates required to assure good inference typically grows faster than the number of variables being measured. Therefore, 100 replicates for 10,000 variables is a scanty data set. Once this supposedly big data set is obtained, it is ‘mined’ by various groping algorithms, usually going under the name of ‘machine learning’ and generally trying to discover patterns in the data. Clustering algorithms cluster data to group together data points that are similar relative to some criterion. Classification algorithms generate classifiers that will then be used to classify future observations. Of course, clustering algorithms form clusters and classification algorithms form classifiers. Whether these clusters and classifiers possess any scientific content is generally not seriously addressed. Some so-called ‘validation index’ may be computed for the clusters and some error estimate might be computed for the classifier, but these are rarely probabilistically justified. Clustering validation indices generally lack any substantiating theory and have been shown to often possess very little correlation with clustering error [39]. Simulation studies have shown small-sample error estimators to typically be inaccurate [29-33]. Moreover, only recently has there been any theoretical analysis of their accuracy [40-43], and this has only scratched the surface. Hence, the scientific literature is littered with thousands of null or erroneous papers referring back and forth to each other in some sort of nihilistic waltz.

This is not to say that data collected without experimental design cannot lead to major discoveries. Perhaps the most salient illustration in this regard is the heliocentric theory. Nicolaus Copernicus used data collected by Claudius Ptolemy about 1,400 years earlier to develop his heliocentric theory, and Johannes Kepler used data collected by Tycho Brahe, who by the way rejected the heliocentric theory, to develop his laws of planetary motion.

It is not that thinking about unplanned data cannot bear fruit; rather, greater progress can typically be achieved by having an idea and then obtaining data directly in response to questions emanating from that idea. Hans Reichenbach states the matter in terms relating to complexity when he writes, ‘An experiment is a question addressed to Nature…. As long as we depend on the observation of occurrences not involving our assistance, the observable happenings are usually the product of so many factors that we cannot determine the contribution of each individual factor to the total result’ [44]. What is the question? This is the question that a scientist must address in his role as a scientist. The more precise the question, the more likely he is to draw from Nature the desired knowledge. According to Arturo Rosenblueth and Norbert Wiener, ‘An experiment is a question. A precise answer is seldom obtained if the question is not precise; indeed, foolish answers – i.e., inconsistent, discrepant or irrelevant experimental results – are usually indicative of a foolish question’ [45]. Finally, let it be noted that Arendt is in full agreement with these assessments. In *The Concept of History: Ancient and Modern*, she states, ‘The natural sciences turned toward the experiment, which, by directly interfering with nature, assured the development whose progress has ever since appeared to be limitless’ [4].

Data mining and Copernicus share a lack of experimental design; however, in contradistinction to data mining, Copernicus thought about unplanned data and changed the world, the key word being ‘thought.’ Copernicus was not an algorithm numerically crunching data until some stopping point, very often with no adequate theory of convergence or accuracy. Copernicus had a mind and ideas. William Barrett writes, ‘The absence of an intelligent idea in the grasp of a problem cannot be redeemed by the elaborateness of the machinery one subsequently employs’ [46]. Or as M. L. Bittner and I have asked, ‘Does anyone really believe that data mining could produce the general theory of relativity’ [17]?

Data mining represents a regression from the achievements of three and a half centuries of epistemological progress to a radical empiricism, in regard to which Reichenbach writes, ‘A mere report of relations observed in the past cannot be called knowledge. If knowledge is to reveal objective relations of physical objects, it must include reliable predictions. A radical empiricism, therefore, denies the possibility of knowledge’ [44]. A collection of measurements together with statements about the measurements is not scientific knowledge, unless those statements are tied to verifiable predictions concerning the phenomena to which the measurements pertain.

One only need read Siddhartha Mukherjee’s *The Emperor of All Maladies: A Biography of Cancer* to be shocked by the suffering inflicted on patients by a radical empiricism. Concerning chemotherapy in the 1970s, he writes,

The NCI meanwhile was turning into a factory of toxins. The influx of money from the National Cancer Act had potently stimulated the institute’s drug-discovery program, which had grown into an even more gargantuan effort and was testing hundreds of thousands of chemicals each year to discover new cytotoxic drugs. The strategy of discovery was empirical – throwing chemicals at cancer cells in test tubes to identify cancer killers – but, by now, unabashedly and defiantly so…

Chemicals thus came pouring out of the NCI’s cauldrons, each one with a unique personality. There was Taxol, one gram purified from the bark of a hundred Pacific yew trees…. Adriamycin,…even at therapeutic doses, it could irreversibly damage the heart. Etoposide came from the fruit of the poisonous mayapple. Bleonmycin, which could scare lungs without warning, was an antibiotic derived from mold.

The greatly expanded coffers of the NCI also stimulated enormous, expensive, multi-institutional trials, allowing academic centers to trot out ever more powerful permutations of cytotoxic drugs. Cancer hospitals, also boosted by the NCI’s grants, organized themselves into efficient and thrumming trial-running machines….

It was trial and error on a giant human scale…. In another particularly tenacious trial, known as the eight-in-one study, children with brain tumors were given eight drugs in a single day. Predictably, horrific complications ensued. Fifteen percent of the patients needed blood transfusions. Six percent were hospitalized with life-threatening infections. Fourteen percent of the children suffered kidney damage; three lost their hearing. One patient died of septic shock…. Most of the children in the eight-in-one trial died soon afterward, having only marginally responded to chemotherapy.

This pattern was repeated with tiresome regularity for many forms of cancer…. Like lunatic cartographers, chemotherapists frantically drew and redrew their strategies to annihilate cancer [47].

This, in the 1970s, after engineers had put men on the moon! What profound ideas lay behind ‘throwing chemicals at cancer cells in test tubes?’

While ignorance of basic scientific method is a serious problem, it is necessary to probe further than simply methodological ignorance to get at the full depth of the educational problem. Science does not stand alone, disjoint from the rest of culture. Science takes place within the general human intellectual condition. Biology cannot be divorced from physics, nor can either be divorced from mathematics and philosophy. One’s total intellectual repertoire affects the direction of inquiry: the richer one’s knowledge, the more questions that can be asked. Schrodinger comments, ‘A selection has been made on which the present structure of science is built. That selection must have been influenced by circumstances that are other than purely scientific’ [48]. If one is intellectually and culturally impoverished, then one’s set of possible selections will be small. Fundamental issues arise in the presence of deep conflicts or inadequacies within scientific theory. Serious study of historical antinomies and their resolutions enriches the mind, provides it with the perspective to see new fundamental issues, and trains it with the ability to think orthogonally to the attacks that have heretofore been thrown against the problem without success.

Can one truly appreciate the present without knowledge of the great past ruptures in human thinking? The Ptolemaic system assured man’s position at the center of the universe until Copernicus put humans on a planet revolving around the sun. From Euclid through Kant, Euclidean geometry provided the framework for human sensibility before this worldview was shattered by the non-Euclidean geometry of Janos Bolyai and Nikolai Lobachevsky. From Aristotle into the eighteenth century, philosophers and scientists accepted a causal world view, even with its bracketing by Galileo and Newton, until David Hume showed with relative ease that there was no logical or empirical support for cause in Nature and mankind was shaken from a comfortable causal, deterministic outlook and tossed into probabilistic insecurity. The Newtonian world of absolute space seemed all too obvious until Einstein shattered the obvious. And from Euclid into the twentieth century, man’s hope for some safe harbor of consistency in his thinking was believed to lie in mathematics until in 1931 Kurt Godel proved that the consistency of any mathematical system rich enough to include whole number arithmetic (which is not much) cannot be proven by the ordinary basic principles of logic. Each of these ruptures was a shock to human understanding and the human position in the universe. All that came before was overturned, and a new human condition came into being. Study of these events, along with the historical and other scientific events surrounding them, forms the intellect. There have been many more disruptive events, perhaps of lesser cosmic import. Some of these pertain to one’s individual scientific pursuits. These, too, need to be placed into historical context so that a student understands what has come before, what the situation is today, and the possibilities of where it will go tomorrow.

Arendt emphasizes that the proper role of education is to raise the child out of the world of children into the adult world. Admittedly, this is a painful process. Nonetheless, it is necessary if the child is to take his place in the adult world and contribute to the maintenance and furtherance of that world. Nothing is more disheartening than to discuss causality and be forced to listen to sophomoric arguments insisting on a causal science. You ask the bright young scientist if he has read Hume’s *An Enquiry Concerning Human Understanding* or Kant’s *Prolegomena to Any Future Metaphysics*, and there is a blank look. Something terribly important for a scientist (or, for that matter, any supposedly educated person) is missing. You are confronting an educational impoverishment that precludes the possibility of a serious discussion and has resulted in the person’s thinking processes being centuries out of date.

Hume and Kant have transformed human reason, and a student must drink that transformation to the dregs or remain intellectually stunted. All that comes after, including twentieth century science, statistics, and engineering, would not be what it is without this transformation. In a few short pages, Hume rocks the scientific and philosophic worlds. Einstein comments, ‘If one reads Hume’s books, one is amazed that many and sometimes even highly esteemed philosophers after him have been able to write so much obscure stuff and even find grateful readers for it. Hume has permanently influenced the development of the best philosophers who came after him’ [49]. William Barrett calls Kant the ‘pivot’ [50]. Barrett provides a diagram in which pre-Kantian rationalism and empiricism enter into Kant and outcome idealism, positivism, pragmatism, and existentialism into the post-Kantian world. There is much here to chew on for the scientist, who is often torn between rationalism and empiricism. After Kant, the phenomena, which are somehow constructed in the mind from sense data, are ever separated from the noumena, which are the things-in-themselves (actual Nature). How one perceives this separation tells much about one’s scientific perspective. As Jose Ortega y Gasset says, ‘Einstein needed to saturate himself with Kant and Mach before he could reach his own keen synthesis. Kant and Mach – the names are mere symbols of the enormous mass of philosophic and psychological thought which has influenced Einstein – have served to liberate the mind of the latter and leave the way open for his innovation’ [51]. Building on the century and a half of philosophic and scientific development beginning with Bacon, Hume and Kant redefine adulthood. To intellectually become an adult, one must walk the path they trod.

When one ponders the massively complex regulatory machinery of the cell, its parallelism, non-linearity, feedback, redundancy, multiple time scales, and stochasticity, it helps immensely to have struggled through Hume’s dismantling of causality and Kant’s analysis of the categories of understanding. A scientific adult views this complex regulatory network in the light of stochastic systems theory, not through the eyes of a child who thinks it possible to gain knowledge of system dynamics by looking at some computer-generated visualization, as if scientific knowledge were somehow akin to gazing at colorful pictures. It is immensely beneficial to have suffered the anguish of maturation in undergraduate school and thereby freed the mind for the non-intuitive peculiarities of a stochastic world, a place in which one’s intuition is constantly shocked. More than that, having broken free of the ‘prejudices’ of the mind and having recognized the mind’s inability to describe Nature in terms of its ordinary physical categories, one learns that story telling has no place in science and that one must stick closely to rigorous mathematical and statistical analysis.

### Playing children’s games

Scientific epistemology has developed so as to formalize quantitative predictive relations between phenomena and to characterize the truth of those relations based upon the efficacy of predictions regarding those phenomena. Not only has this epistemology led to a grounding of scientific knowledge that overcomes the skepticism of Hume, it has also resulted in mankind’s ability to alter the course of Nature in ways beneficial to human existence.

Peering into a future ubiquitous with data mining and the oxymoronic data science, troubling questions arise. How many patients will be improperly treated based on gene- or protein-based diagnostic tests developed using statistically meaningless performance estimates? How many billions of dollars will be wasted on studies so poorly designed that they cannot possibly produce useful results? How many petabytes of unstructured data will be generated by academic centers to be groped through in mindless darkness? It all sounds utterly childish to those who have walked the epistemological path from Aristotle to Galileo to Newton to Hume to Kant to Einstein to Schrodinger. Oblivious to the demands of science, the educationally impoverished proponents of this latest incarnation of radical empiricism are playing children’s games, except that these games will not pay off in candy, but in human suffering on a grand scale - even if it is only the result of the billions of wasted dollars that could have been spent on serious research.

What has brought our civilization to this point? Again we turn to Arendt: ‘In education this responsibility for the world takes the form of authority…. Authority has been discarded by the adults, and this can mean only one thing: that the adults refuse to assume responsibility for the world into which they have brought the children’ [2]. Irresponsibility has led to the impoverishment of education and a consequent loss of scientific capability. One might laugh at the ignorance that Mukherjee repeatedly highlights, but the suffering of innocent patients at the hands of those whose reasoning lies somewhere in the fifteenth century is not a laughing matter. More recently, the world witnessed a bizarre fiasco in the Gulf of Mexico, where we were assured that the ‘best’ engineers were on hand to stop an oil spill. These ‘best’ engineers demonstrated their fifteenth century capability by pouring rocks into a hole and surrounding floating oil with poles dragged by ships, something that Odysseus might have done. The ludicrousness of the whole operation can easily be seen by comparing this woeful episode with the engineering operations run by Robert Oppenheimer and Werner von Braun.

The plight of science is not a scientific problem. It lies outside of science, in a general collapse of authority. Ortega y Gasset places the matter in the wider context:

Whoever wishes to have ideas must first prepare himself to desire truth and to accept the rules of the game imposed by it. It is no use speaking of ideas when there is no acceptance of a higher authority to regulate them, a series of standards to which it is possible to appeal in a discussion. These standards are the principles on which culture rests. I am not concerned with the form they take. What I affirm is that there is no culture where there are no standards to which our fellow-men can have recourse…. Barbarism is the absence of standards to which appeal can be made [51].

The scientific epistemology posits standards developed over centuries to ground knowledge with a functional, phenomenal, and inter-subjective concept of truth. The theory can be understood by anyone possessing the requisite mathematical knowledge. The experimental protocols can be understood by anyone possessing knowledge of the experimental apparatus. The operational definitions, corresponding statistics, and validation criteria take logical or mathematical form and again can be understood by anyone possessing the requisite knowledge. The overall theory, mathematical and experimental, is therefore inter-subjective. This is not to say that two people cannot disagree on whether to accept a theory. That will depend on their validation criteria. One may impose stronger, or different, validation criteria than the other. There is inter-subjectivity because each understands the other’s criteria. This is not to say that the truth of the theory is universally applicable. On the contrary, it is constrained by the context in which the relations (equations) of the theory are purported to hold. Outside that context, the relations might fail, so that the context in which the operational definitions can be applied must be specified. All of this provides the standards by which the higher authority is constituted. But that authority must be manifested by human beings, and these must be sufficiently educated so that they can make judgments in accordance with that authority. If educators fail in their responsibility to educate, then the higher authority becomes vacuous because in practice there will be no one, or an insufficient number, to exercise it.

If educators fail to educate, then civilization, not just science, is at grave risk. Will Durant, who spent his life studying the rise and fall of civilizations, puts the matter starkly: ‘For civilization is not something inborn or imperishable; it must be acquired anew by every generation, and any serious interruption in its financing or its transmission may bring it to an end. Man differs from the beast only by education, which may be defined as the technique of transmitting civilization’ [52]. And what is the relationship of science to civilization? Perhaps this question can best be answered by noting that Will and Ariel Durant list three books as ‘the basic events in the history of modern Europe’: *Philosophiae Naturalis Principia Mathematica* (Isaac Newton), *De Revolutionibus Orbium Coelestium* (Nicolaus Copernicus), and *The Origin of the Species* (Charles Darwin) [53]. For Will and Ariel Durant, these books are not simply the basic events of science; they are the fundamental events that have driven the overall philosophic, religious, and political evolution of Western Civilization - more profound than Martin Luther nailing his 95 theses to the door of the Castle Church in Wittenberg, than Renes Descartes’ systematic doubt, than Jean-Jacques Rousseau and the French Revolution. To appreciate the monumental roles of Copernicus, Newton, and Darwin, one must be able to place and understand them in the historic stream of philosophic thought. To bring students into the university and leave them ignorant of Plato, Aristotle, Bacon, Hume, Kant, et al*.* is to deny them a meaningful education. It is to leave them outside the course of civilization and to stunt their growth into intellectual adulthood. In Arendt’s words, it is to ‘strike from their hands their chance of undertaking something new, something unforeseen by us’. And the horrific cost will be borne by future generations.

Once lost or seriously diminished, modern science will not be easily resuscitated. It is a unique and precious gift to our civilization, one whose continuance is not guaranteed: Ortega y Gasset recognizes its tenuous character when he writes,

Has any thought been given to the number of things that must remain active in men’s souls in order that there may still continue to be ‘men of science’ in real truth?… Experimental science is one of the most unlikely products of history. Seers, priests, warriors and shepherds have abounded in all times and places. But this fauna of experimental man apparently requires for its production a combination of circumstances more exceptional than those that engender the unicorn. Such a bare, sober fact should make us reflect on the supervolatile, evaporative character of scientific inspiration [51].

Whatever ‘must remain active in men’s souls’, to a great extent it must come from educators who recognize that it is not their duty to make children happy; rather, it is their duty to transform children into adults.

## Conclusions

Transforming children into adults - this has been the theme of this essay and it is also the theme of Arendt’s *The Crisis in Education*, in which she notes, ‘Childhood is a temporary stage, a preparation for adulthood’. This essay is not about lack of innate intelligence or a lack of desire to accomplish great things. A student may enter the academy with both a brilliant mind and a longing to join the community that has driven the great scientific enterprise, but if the academy shirks its responsibility and impoverishes that student, then that brilliant and ambitious mind will not come close to achieving its true potential. The manner in which human beings scientifically perceive the world has gone through at least four radically transforming periods that can be marked by certain names: (1) Plato and Aristotle, (2) Galileo and Newton, (3) Hume and Kant, and (4) Einstein and Heisenberg. Here we are not talking about radical theories, but rather radical transformations of mind. In that sense, these are maturing transformations, in each case the notion of intellectual adulthood being redefined. The young student enters the academy as a wet-behind-the-ears babe and must be transformed through these stages, perhaps kicking and screaming, into an adult who appreciates the road humans have traveled in two and half millennia to achieve the current state of maturity. Only then does the aspiring scientist appreciate the limitations of science and the mathematical, logical, and experimental rigor necessary to achieve scientific truth.

## Competing interests

The author declares that he has no competing interests.
